# The impact of perceived environmental competitiveness on employee mental health: a moderated mediation model of job crafting and work–family conflict

**DOI:** 10.3389/fpubh.2024.1433215

**Published:** 2024-08-16

**Authors:** Sheng Cheng, Yumei Wang

**Affiliations:** School of Business, Suzhou University of Science and Technology, Suzhou, China

**Keywords:** perceived environmental competitiveness, job crafting, mental health, work–family conflict, moderated mediation model

## Abstract

Drawing from the conservation of resources theory, this study proposes that individuals who perceive environmental competitiveness may improve their mental health through their job crafting behaviors at work. Data were collected from 450 full-time Chinese employees using a three-wave time-lagged approach. The results showed that perceived environmental competitiveness is positively correlated with job crafting, and job crafting has a positive relationship with mental health. Moreover, the results indicated that job crafting mediates the relationship between perceived environmental competitiveness and mental health. Additionally, the present study found that work–family conflict plays a moderating role in the relationships among environmental competitiveness, job crafting and mental health. A moderated mediation model was proposed in this study. Finally, theoretical and practical implications of this study are also discussed.

## 1 Introduction

In recent years, occupational health and safety has garnered increasing attention from researchers (1, 2). It has been argued that organizations should not only care about employees’ physical health and safety in the workplace but should also place equal importance on protecting and enhancing their psychological well-being at work and mental health beyond the workplace (3–6).

In the workplace, a competitive work environment is frequently regarded as a source of stress, which can not only have impacts on employees’ work behaviors, outcomes in the workplace, but also can have effects on employees’ mental health beyond the workplace. Previous studies have indicated that employees who perceive a competitive environment in the workplace may experiencing negative effects such as increased knowledge hiding behaviors (7), lower job satisfaction, and higher job stress (8). These influences can also extend beyond the workplace, continuing to influence employee mental health through spillover effects (9). However, a competitive work environment can also produce some positive outcomes. Previous study indicated that employees who perceive a competitive environment in the workplace may lead to employees exhibit more proactive work behaviors, which in turn, increase employees’ organizational commitment and organizational performance (10). Based on these arguments, the present study posits environmental competitiveness has a double-edged effect, understanding how to convert environmental competitiveness into motivation for employees thereby creating positive effects on employees, is a worth area of exploration. In other words, this research aims to addresses the gap in existing literature by using conservation of resources (COR) theory (11) to identifying the mechanisms through which employees’ perception of environmental competitiveness can have positively impacts on employees’ mental health.

Base on the resources acquisition perspective of COR theory, the present study hypothesizes that, in a resource-constrained society, competition is the swiftest and most effective way for individuals to acquire, master, and utilize diverse resources in their work, meeting their needs to achieve their goals (12). Therefore, when employees perceive environmental competitiveness in the work domain, they may realize there are additional opportunities and resources can be obtained through interpersonal competition, including promotion prospects, training resources, and project assignments. Being aware of these additional opportunities and resources may positively impact employees’ motivation, driving them to seek further resources, challenges, and reduce job demands. These behaviors were called job crafting (13). Following this, when employees achieve success in crafting their jobs, the sense of accomplishment also improves their mental health in non-work domains through a spillover effect (5, 14). Specifically, the present study also hypothesizes that job crafting may mediate the relationship between perceived environmental competitiveness and mental health.

Furthermore, the present study proposes that resources in employees’ non-work domains may also influence their work domain through a spillover effect. Therefore, this study examines the boundary conditions of the relationships among perceived environmental competitiveness, job crafting, and mental health within the context of work–family dynamics, specifically focusing on moderating role of work–family conflict. We expect that such a moderated mediation model can effectively explain the spillover mechanisms among employees’ perception, work behaviors, and mental health in their work and non-work domains.

**Figure 1:**
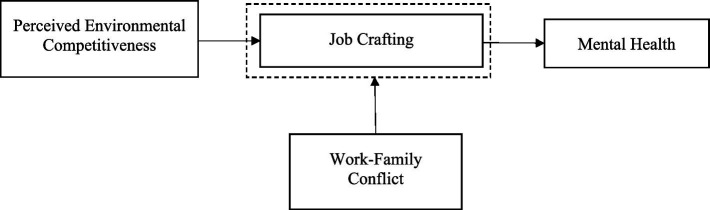
The proposed conceptual scheme.

Finally, we also hope to identify effective interventions to leverage the positive impact of environmental competitiveness and mitigate its negative effects. Figure 1 shows the conceptual model of the present study.

## 2 Theory and hypotheses

### 2.1 Relationship between perceived environmental competitiveness and job crafting

Perceived environmental competitiveness refers to the degree to which individuals perceive the intensity and tension of competition in their current environment (15, 16). From the COR theory perspective, Hobfoll (11) suggested that individuals strive to obtain, retain, foster and protect the resources that they value the most. However, environmental competitiveness in the workplace can be interpreted as two separate social cues through social information processing (17): (1) limited resources in organization are distributed based on personal efforts and achievement; and (2) personal resources can be contested by others. These social cues motivate individuals to seek additional resources and avoid the threat of personal resource loss. Therefore, when individuals perceive competitiveness in their current environment, it suggests that the present resources of their organizations are limited and unevenly distributed. In such a scenario, individuals would proactively seek additional resources and try to protect their existing resources through their personal extra efforts or achievement that surpass others (12, 18). Previous studies have identified that both striving for additional resource gains and preventing resource from loss may motivate individuals to exhibit more positive attitudes and behaviors at work (19–22). Building on these previous findings, this study suggests that perceived environmental competitiveness within an organization does not impose burdens on individuals but can act as a driving force, motivating individuals to work harder, such as job crafting.

Job crafting refers to employees proactively modify the boundaries and conditions of job-related tasks and relationships to improve the meaning, engagement, and satisfaction they receive from their work domain (13, 20, 23). Based on COR theory, the perception of environmental competitiveness implies resources limitation within organization, and this motivates employees to proactively strive for resources they value. Therefore, they would maximize resource utilization through effective self-management and optimization (24–26). Specifically, employees may streamline or optimize their work processes to reduce demands (27), seek valuable suggestions to enhance efficiency (28), participate in training programs to improve competency (29), and embrace more challenges to achieve a sense of accomplishment (30). These proactive behaviors align with the concept of job crafting.

Moreover, the perception of environmental competitiveness also implies the potential threat of losing existing resources. According to COR theory, when employees perceive threats to their existing resources, they are likely to adapt their working strategies to meet the demands of the changing work conditions (26, 31, 32). These adaptations may include employees proactively adjusting and redesigning their job tasks, responsibilities, and relationships to enhance their performance. For example, employees may actively improve their skills and capabilities to meet job demands and challenges effectively (33), enhance collaboration with team members to improve overall team performance (34), and expand their professional social networks to increase their social capitals (35). These behaviors also consistent with the concept of job crafting. Thus, we propose that perceived environmental competitiveness increases job crafting. The hypothesis is as follow:

*H1:* Perceived environmental competitiveness is positively correlated with job crafting.

### 2.2 Relationship between job crafting and mental health

Mental health refers to the state of psychological well-being in which an individual can cope with the normal stresses of life (36). It is a psychological state characterized by individual resilience, self-esteem, and the capacity to enjoy life (37). Beyond uncontrollable factors like biological elements and family history, the most significant influence on mental health is life experiences (38). Therefore, we propose that employees’ mental health is affected by job crafting in workplace through spillover effects.

Spillover refers to a cross-domain transmission of resources from one area of an individual’s life to another (14). In this context, the present study posits that, through the spillover effect, employees leverage the resources acquired via job crafting to enhance their mental health. First, seeking resource of job crafting provides individual with additional personal resources, such energy, time and skills. Seeking resources is when an employee proactively seeks advice from colleagues and supervisors, actively acquires new skills or knowledge, and purposefully asks for help when facing difficulties (20, 23). These behaviors not only help individuals save time and energy at work but also enhance their positive psychological states or emotions by solving difficulties (13, 39, 40). Therefore, by increasing their efficiency and enhancing their positive affects at work, through the spillover effect, this also improves their mental health in non-work domains (41). Additionally, job crafting motivates employees to learn new skills and knowledge in the workplace, these acquired skills and knowledge also can help people to conquer life challenges not linked to their jobs, further enhancing their role acting in other non-work domain via spillover effect (14). Second, seeking challenges through job crafting can induce a sense of accomplishment. Seeking challenges refers to individuals proactively asking for more difficult tasks and increased responsibilities; the sense of accomplishment is produced through their successful completion (20, 23, 42, 43). This positive feeling further nurtures their mental health through the spillover effect (5). Finally, reducing demands through job crafting alleviates some of the mental and physical stress experienced at work. Reducing demands involves employees simplifying tasks to alleviate emotional, mental, and physical tensions (20, 23). By reducing demands, employees do not need to draw on additional resources from other domains to cope with work stress (44, 45). Moreover, the personal resources saved by reducing demands of job crafting, may also improve their mental health in non-work domains through the spillover effect. In this context, we present the following hypothesis:

*H2:* Job crafting is positively correlated with mental health.

### 2.3 The mediating role of job crafting

This study further posits that job crafting plays a mediator role between perceived environmental competitiveness and mental health. According to COR theory, when individuals perceive environmental competitiveness, they interpret the phenomenon through social information processing as two distinct social cues (additional resource obtaining and existing resources protecting) (11). However, both types of social cues can motivate individuals to engage in job crafting. Following this, as individuals succeed in job crafting, the extra personal resources and positive affects produced further enhances their mental health through the spillover effect. Hence, we also hypothesize:

*H3:* Job crafting mediates the relationship between perceived environmental competitiveness and mental health.

### 2.4 The moderating role of work–family conflict

The positive effect of perceived environmental competitiveness on job crafting can be increased by clearly identifying boundary conditions. Drawing on COR theory (11) and the spillover effect (41), the present study further suggests that work–family conflict is a potential moderating factor and impacts the relationship between perceived environmental competitiveness and job crafting.

Work–family conflict arises when an individual faces increased challenges in their family role due to concurrent engagement in the work role (46). This challenge is characterized by time-, strain-, and behavior-based conflicts (47–49). However, to resolve work–family conflict requires individual to spend additional personal resources on family domain (50, 51). Based on these foundations, this study posits that the interaction between work–family conflict and perceived environmental competitiveness has a moderating effect on job crafting from a resource perspective. Specifically, individuals with low work–family conflict have more personal resources with which to face environmental competitiveness and therefore exhibit more job crafting behaviors than those with high work–family conflict.

First, individuals with low work–family conflict have fewer time-based conflicts (50). Having more time-based resources provides them with greater time-based flexibility, and their time-based resources can be transferred to their work domain (9). Therefore, individuals with low work–family conflict have more time-based resources to engage in job crafting when facing the environmental competitiveness. In contrast, individuals with high work–family conflict have more time-based conflicts (46), leaving them with limited spare time-based resources to transfer to the work domain (51). Hence, when confronted with environmental competitiveness, they are less likely to demonstrate additional job crafting behaviors.

Second, individuals with low work–family conflict have fewer strain-based conflicts, and have more positive resources, such as spousal support (46). The positive effects can spillover into individuals’ work domain and generate positive emotions in their work (41). Therefore, individuals with higher emotional resources exhibit higher job crafting motivation. In contrast, individuals with high work–family conflict have more strain-based conflicts (50), meaning that they have fewer positive emotional resources (46). Therefore, the limited emotional resources can spillover into their work domain, consequently would not enhance their motivation for job crafting.

Finally, individuals with low work–family conflict may also have low behavior-based conflicts, so in their family relationships they can easily express warmth, emotional connection, and vulnerability (46). In other words, such individuals need fewer personal resources to fake their true psychological states and emotions (9). Therefore, because they have many personal resources that can be transferred to their work domain to exhibit many job crafting behaviors when in times of perceived environmental competitiveness. Conversely, individuals with high work–family conflict may have high behavior-based conflict, so they need a lot of personal resources to interact well with family members (46). In this situation, such individuals lack the flexibility of superfluous personal resources to bring into their work domain. Thus, individuals with high behavior-based conflict may present additional job crafting behaviors when facing environmental competitiveness.

Based on the above discussion, we present the following hypothesis:

*H4:* Work–family conflict moderates the positive relationship between perceived environmental competitiveness and job crafting, such that the effect of perceived environmental competitiveness on job crafting is stronger when work–family conflict is low.

This study also posits that work–family conflict moderates the mediation relationship between perceived environmental competitiveness and mental health through job crafting. Specifically, individuals with low work–family conflict have more personal resources to transfer to their work domain through the spillover effect, leading to additional job crafting behaviors. This, in turn, ultimately enhances their mental health. In contrast, individuals with high work–family conflict have few additional personal resources to transfer to their work domain. Therefore, they would not present additional job crafting behaviors, and their mental health is also not improved via the spillover effect. Therefore, the present study hypothesizes:

*H5:* Work–family conflict moderates the mediated effect of job crafting on the relationship between perceived environmental competitiveness and mental health, such that the mediated effect is stronger when work–family conflict is low.

## 3 Methods

To prevent common method biases (52) and to ensure the external validity of the present study, data was collected in three waves from a variety of organizations in China. The study also invited four bilingual scholars with backgrounds in psychology and management to monitor the back-translation process, based on Brislin (53). This approach was employed to reduce potential biases that could arise during the translation of the original English scales into Chinese. All scales used a six-point Likert-type scale, ranging from 1 (strongly disagree) to 6 (strongly agree).

### 3.1 Participants and procedures

In this study, we employed a convenience sampling method. We contacted human resources departments of organizations that had established collaborations with the researchers to distribute the questionnaires. The human resources departments confirmed that selected participants did not have a history of mental illness. In these samples, participants were from various industries (such as high-technology, manufacturing, education, healthcare, finance, retails, and information technology), different geographic locations in China, and different organizations sizes. Therefore, these diversities ensure the randomness and representativeness of the sample.

Moreover, to enhance questionnaire response rates among participants, we established a lottery mechanism. Participants who successfully completed all three waves of the questionnaire had the opportunity to win a $100 USD supermarket gift voucher. The lottery mechanism can increase response rates because it appeals to participants’ interest in potentially winning a prize, which encourages more individuals to engage with the survey. Additionally, the mechanism may attract participants who might not otherwise have participated, thereby increasing the sample size and improving the statistical validity of the study. Additionally, the last six digits of participants’ phone numbers were used as unique identification codes for each wave of online anonymous questionnaires. In Wave 1, we distributed nearly 900 questionnaires and collected 724 valid responses, giving a response rate of 80.44%. After 1 month, in Wave 2, we sent questionnaires to those participants who had completed the Wave 1 questionnaires, and received 573 valid responses. The response rate of wave two was 79.14% Finally, 1 month after Wave 2, we distributed the Wave 3 questionnaires to participants who had successfully completed both the questionnaires in Wave 1 and 2. In Wave 3, 450 valid responses were collected and the response rate was 78.53%.

The demographics of the participants were as follows: 172 were males (38.22%), 278 were females (61.78%); the majority were 31–40 years old (295, 65.56%), 106 were 18–30 years old (23.56%), and rest were over 41 years old (49, 10.88%); 267 had a bachelor’s degree (59.33%), and 131 had postgraduate qualifications (29.11%); 200 were general staff (44.44%), 106 were junior management (23.56%), 97 were mid-level management (21.56%), and 47 were senior management (10.44%); 222 had 11–20 years of tenure (49.33%), 131 had 5–10 years of tenure (29.11%), 66 had 0–5 years of tenure (14.67%), and 31 had over 20 years of tenure (6.89%).

### 3.2 Measures

#### 3.2.1 Perceived environmental competitiveness (wave 1)

Perceived environmental competitiveness was measured in Wave 1 with a four-item scale developed by Jansen et al. (18). A sample item is “Competition in our organization is intense.” The Cronbach’s α of perceived environmental competitiveness measurement in this study is 0.90.

#### 3.2.2 Job crafting (wave 2)

Job crafting was captured using a 13-item scale developed by Petrou et al. (20). A sample item of job crafting scale is “When I have difficulties or problems at my work, I discuss them with people from my work environment.” The Cronbach’s α of job crafting scale in this study is.86.

#### 3.2.3 Mental health (wave 3)

The 12-item General Health Questionnaire (GHQ-12) developed by Goldberg and Williams (36) was employed to measure individual’s mental health in Wave 3. In this measure, high scores indicate positive mental health. A sample item is “I am able to concentrate on what I am doing.” The Cronbach’s α of GHQ-12 in this study is 0.88.

#### 3.2.4 Work–family conflict (wave 3)

Work–family conflict was also collected in Wave 3 by 5-item developed by Gutek et al. (54), a sample item of work–family conflict scale is “My family dislike how often I am preoccupied with my work while I am at home.” The Cronbach’s α of work–family scale in this study is 0.83.

#### 3.2.5 Control variables (wave 1)

The present study collected demographic variables including gender, age, educational level, job position, and job tenure in Wave 1. These variables were chosen as common control variables because they can significantly impact individual perceptions, behaviors, and outcomes in the workplace.

## 4 Results

Table 1 demonstrates the means, standard deviations (SD), bivariate correlations, and Cronbach’s α values of all assessed variables. In Table 1, the correlations shown indicate that perceived environmental competitiveness is positively associated with job crafting (*r* = 0.26, *p* < 0.001). There is also a positive correlation between job crafting and mental health (*r* = 0.20, *p* < 0.001). Therefore, Hypotheses 1 and 2 are supported based on the calculated correlation coefficients.

**Table 1:** Variables means, standard deviations, reliabilities, and correlations (*N* = 450).

Variables	Mean	SD	1	2	3	4	5	6	7	8	9
Gender†	1.62	0.49									
Age†	2.85	0.68	−0.08								
Education†	5.15	0.69	−0.05	0.00							
Position†	1.98	1.04	−0.18^***^	0.37^***^	0.14^**^						
Organizational tenure†	3.46	0.87	−0.03	0.74^***^	−0.00	0.45^***^					
Perceived environmental competitiveness	3.76	1.13	−0.08	0.06	−0.02	0.10^*^	0.13^**^	(0.90)			
Job crafting	4.44	0.62	−0.08	0.08	0.04	0.17^***^	0.15^**^	0.26^***^	(0.86)		
Mental health	4.44	0.76	−0.01	0.11^*^	0.05	0.17^***^	0.13^**^	0.03	0.37^***^	(0.88)	
Work–family conflict	3.28	1.04	−0.12^*^	0.00	0.03	−0.01	0.01	0.20^***^	0.06	−0.40^***^	(0.83)

Internal consistency (Cronbach’s alpha, *α*) are presented in the diagonal.

Gender (1 = male, 2 = female); Age (1 = 18–25 years old, 2 = 26–30 years old, 3 = 31–40 years old, 4 = 41–50 years old, 5 = 50–60 years old, 6 = 60 years old above); Education (1 = primary school or below, 2 = high school, 3 = (vocational) senior high school, 4 = junior college; 5 = bachelor’s degree, 6 = postgraduate or above); Position (1 = staff, 2 = junior manager, 3 = mid-level manager, 4 = senior manager); Organizational tenure (1 = 0–1 years, 2 = 1–5 years, 3 = 6–10 years, 4 = 11–20 years, 5 = 20 years above).

*p < 0.05; **p < 0.01; ***p < 0.001.

### 4.1 Model analyses

The model comparison results are presented in Table 2. Confirmatory factor analysis with parceling rules (55) was employed to test the model fit of our hypothesized model with collected data using AMOS 21 software. The overall results of the confirmatory factor analysis shown in Table 2 indicate that the four-factor hypothetical model demonstrated the best goodness-of-fit with the data [*x^2^* (59) = 225.84; comparative fit index (CFI) = 0.94; Incremental Fit Index (IFI) = 0.94; Tucker–Lewis index (TLI) = 0.93; root mean square error of approximation (RMSEA) = 0.08]. The present study also tested four other alternative-factors models. The models’ goodness-of-fit statistical results (as shown in Table 2) indicate that the hypothetical model with four factors had the best model fit for data.

**Table 2:** Results of confirmatory factor analyses of the measures (*N* = 450).

Model	Factors	*χ*^2^	df	△*χ*^2^/df	CFI	IFI	TLI	RMSEA
Hypothetical Model	4 factors	225.84	59		0.94	0.94	0.93	0.08
Model 1	3 factors	414.24	62	62.80	0.88	0.88	0.85	0.11
Model 2	3 factors	486.48	62	86.88	0.86	0.86	0.82	0.12
Model 3	2 factors	1086.69	64	172.17	0.66	0.66	0.58	0.19
Model 4	1 factor	1745.57	65	253.29	0.43	0.44	0.32	0.24

Bootstrap sample size = 50,000.

Hypothetical Model (4 factors, perceived environmental competitiveness; job crafting; mental health; work–family conflict).

Model 1 (3 factors: perceived environmental competitiveness; job crafting and mental health merged; work–family conflict).

Model 2 (3 factors: perceived environmental competitiveness and job crafting merged; mental health; work–family conflict).

Model 3 (2 factors: perceived environmental competitiveness, job crafting and work–family conflict merged; mental health).

Model 4 (1 factor: all variables are merged into one factor).

### 4.2 Hypothesis testing

To test the direct effects of Hypothesis 1 and Hypothesis 2, we employed the PROCESS macro for SPSS, developed by Hayes (56). The PROCESS macro is an analysis software includes hundreds of model syntaxes, allowing researchers to test complex relationships among variables. Hypothesis 1 states that there is a positive correlation between perceived environmental competitiveness and job crafting. After controlling for the demographic variables, the results of direct effect using linear regression in the PROCESS macro indicated that perceived environmental competitiveness was found to have a significant positive relationship with job crafting (*β* = 0.13, SE = 0.03, *p* < 0.001). Hence, Hypothesis 1 is supported. Similarly, Hypothesis 2 states that job crafting is positively related to mental health. The results of direct effect using linear regression in the PROCESS macro reveal that there is a strong positive correlation between job crafting and mental health (*β* = 0.45, SE = 0.06, *p* < 0.001). Therefore, Hypothesis 2 is statistical supported.

**Figure 2:**
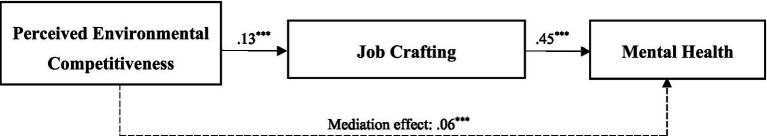
Results of the hypothetical mediation model. *N* = 450. Standardized regression coefficients are reported. Bootstrap sample size = 50,000. χ^2^ (32) = 107.95; CFI = 0.97; TLI = 0.95; IFI = 0.97 RMSEA = 0.07. ^***^p < 0.01.

We also examined the indirect effect of Hypothesis 3 using the existing mediation model syntax and the Sobel test (57) in the PROCESS macro (56). Hypothesis 3 posits that job crafting mediates the relationship between perceived environmental competitiveness and mental health. The results of the Sobel test show that job crafting plays a mediator role between perceived environmental competitiveness and mental health (indirect effect = 0.06, SE = 0.01, *t* = 4.28, *p* < 0.001). Moreover, we further test the mediating effect using 50,000 bootstrap samples with both structural equation modeling in the AMOS 21 and the existing mediation model syntax in the PROCESS macro. The results indicate that the 95% confidence intervals (95% CI) of the mediating effect is [0.03, 0.09]. Thus, Hypothesis 3 is supported. Figure 2 shows the direct, indirect, and mediated effect of Hypotheses 1, 2, and 3.

**Figure 3:**
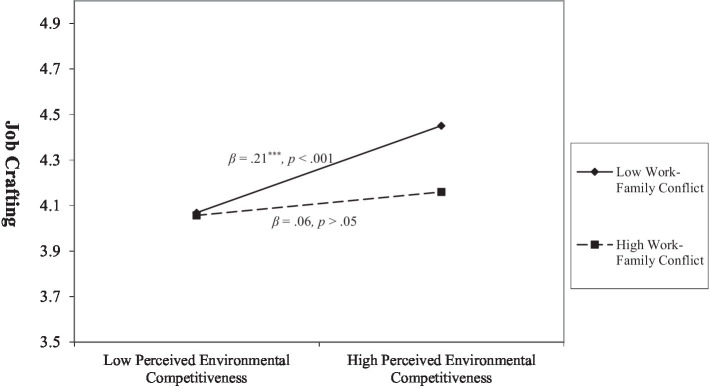
Moderating effect of work–family conflict on the relationship between perceived environmental competitiveness and job crafting.

Hypothesis 4 states that the positive relationship between perceived environmental competitiveness and job crafting is moderated by work–family conflict. The existing simple moderation model syntax in the PROCESS macro was employed to test the simple moderating effect of Hypothesis 4. As Table 3 shows, the interaction effect of work–family conflict and perceived environmental competitiveness was significant [*β* = −0.07, SE = 0.02, 95% CI = (−0.12, −0.03)]. Further, we explored the moderating effect by categorizing work–family conflict into three levels to represent moderator at low (−1 SD), mean, and high (+1 SD) levels. As shown in Table 3, the moderating effects of work–family conflict only had statistical significance at low [*β* = 0.21, SE = 0.03, 95% CI = (0.14, 0.28)] and mean [*β* = 0.13, SE = 0.03, 95% CI = (0.08, 18)] levels. Figure 3 shows the moderating effect of work–family conflict on the relationship between perceived environmental competitiveness and job crafting. Hence, Hypothesis 4 is supported.

**Table 3:** Regression results for moderation (*N* = 450).

Values of work–family conflict in simple moderated effect for job crafting				
**Work–family conflict**	***b***	***SE***	Boot LL 95% CI	Boot UL 95% CI
−1 SD (2.24)	0.21	0.03	0.14	0.28
*M* (3.28)	0.13	0.03	0.08	0.18
+1 SD (4.32)	0.06	0.03	−0.01	0.12
**Index of simple moderated**	−0.07	0.02	−0.12	−0.03
Work–family conflict moderated mediation results for mental health				
−1 SD (2.24)	0.10	0.04	0.06	0.14
*M* (3.28)	0.06	0.03	0.03	0.09
+1 SD (4.32)	0.03	0.03	−0.01	0.06
**Index of moderated mediation**	−0.03	0.01	−0.06	−0.01

Unstandardized regression coefficients are reported.

Bootstrap sample size = 50,000.

LL = lower limit; CI = confidence interval; UL = upper limit.

Hypothesis 5 further predicted work–family conflict moderates the mediation effect of job crafting on the relationship between perceived environmental competitiveness and mental health. The moderated mediation effect was also tested by using the existing simple moderated mediation model syntax in the PROCESS macro, and the results were presented in Table 3. The index of moderated mediation received a significance in statistical analyses [*β* = −0.03, SE = 0.01, 95% CI = (−0.06, −0.01)]. Moreover, similar as simple moderating effect of Hypothesis 4, the moderated mediating role of conflict only received significances at the low level [*β* = 0.10, SE = 0.04, 95% CI = (0.06, 14)] and mean level [*β* = 0.06, SE = 0.03, 95% CI = (0.03, 09)]. Therefore, Hypothesis 5 is statistically supported.

## 5 Discussion

The present study employed the insights of COR theory and spillover effect to examine the relationships among perceived environmental competitiveness, job crafting, mental health, and work–family conflict. The results show that there is a positive correlation between perceived environmental competitiveness and job crating. Moreover, job crafting is positively correlated with mental health, and job crafting plays a mediating role between perceived environmental competitiveness and mental health. Finally, the confirmed the moderating role of work–family conflict; specifically, individuals with low work–family conflict exhibit more job crafting behaviors and have better overall mental health than those with high work–family conflict.

### 5.1 Theoretical implications

The present study makes four theoretical contributions to the literature. First, this study contributes to perceived environmental competitiveness literature by identifying its positive effect on job crafting. Moreover, this study applies COR theory to investigate how individuals interpret environmental competitiveness. We argued that in this context, employees use social cues to gain or protect resources from loss, and can transfer non-work domain resources to their work to be used in job crafting. Theoretically, this study is in line with previous studies that found that environmental competitiveness can not only be regarded as a stressor at work (8) via resource loss perspective (24) but also can be regarded as a motivator to obtain additional resources (58). Nevertheless, according to COR theory (11), whether viewed from the perspective of resource gain or loss, perceived environmental competitiveness motivates individuals to work harder to acquire additional resources or protect existing resources.

Second, the present study contributes to the literature on spillover effect, job craft, and mental health. Prior studies have mainly focused on the spillover effect within work and family domains (9, 41, 45), with limited research addressing the spillover effect between work and life domains. This study shows that the spillover effect can also occur within work and non-work domains and there is a positive correlation between job crafting and mental health. Taken together, prior findings and our results indicate that the personal resources generated by job crafting not only have positive effect on work performance (40), work engagement (28), and job satisfaction (39) but they also have positive impacts on an employee’s non-work domain—thereby improving their mental health in other areas of life. Therefore, the current study moves the job crafting literature beyond its previous boundaries and into the research field of non-work domains.

Third, this study examined the underlying mechanism of the effect of perceived environmental competitiveness on mental health through job crafting. The results showed that job crafting plays a mediator role in the relationship between perceived environmental competitiveness and mental health. According to COR theory (11) and social information processing (59), when individuals perceive environmental competitiveness, they interpret it into two ways: a need to gain additional resources and to protect existing resources. However, these two social cues can motivate individuals to “fight” for resources by engaging in job crafting behaviors. Subsequently, the personal resources gained and the positive affect generated through job crafting not only assist individuals in better task completion but also positively enhance their mental health through the spillover effect. Therefore, our study reveals the process through which individuals transform the impact of workplace environment into work motivation and behaviors, ultimately promoting personal well-being. This provides new insights for future studies in the work–life interface.

Finally, the present study also contributes to the work–family conflict literature by exploring the moderating effects of work–family conflict. Previous studies mainly focused on the antecedents and consequences of work–family conflict (48, 60–62), little research has investigated the interaction between work–family interface and the work context. Thus, this study not only identified work–family conflict as a boundary condition of the relationship between perceived environmental competitiveness and job crafting, but it also provides a novel insight into how work–family conflict can serve as a moderator in the relationship between work-family interface and work context.

### 5.2 Practical implications

The present study also has several practical implications for managers. Firstly, creating a sense of appropriate competition intensity for employees is beneficial. Although such a work environment can increase work-related stress (8), employees tend to transform this pressure into motivation to complete tasks (7). However, the intensity of competition should not be excessive, as hyper-competition can lead to higher turnover intention (63). Previous research had provided several strategic interventions. For instance, organizations and managers should set clear and achievable performance expectations aligned with organizational goals (64). Moreover, organizations and managers should implement fair reward systems based on performance to stimulate healthy competition (7). Furthermore, fostering a culture of collaborative competition is also important, which could establish a win-win competitive environment (65). Therefore, managers may simultaneously increase healthy competition while fostering a collaborative and supportive work environment with clear guidelines on fair resource distribution.

Secondly, when a competitive atmosphere arises within an organization, managers should provide appropriate guidance to encourage employees to engage in job crafting. For example, organizations and managers can empower employees more autonomy to shape their roles and tasks (27, 66), provide support and opportunities for employees to acquire new skills and knowledges (20), and allow flexibility in work arrangements (67, 68). Moreover, conducting job crafting workshops and training are also effective strategies to educate employees on job design knowledges and skills (23). Additionally, organization and managers should establish regular feedback and reward system to acknowledge and reward employees who proactively redesign their jobs and roles to align with organizational goals (13). By doing so, employees not only improve their work performance (40), engagement (28) and satisfaction (39) through job crafting, but they also bring the personal resources and positive affects generated by job crafting into their personal lives through the spillover effect, ultimately enhancing their mental health.

Finally, emphasizing employees’ work–family balance also acts as a catalyst to enhance employee work efficiency. Previous studies have provided several strategies to enhance employee work-family balance. For instance, organizations and managers should implement more flexible work policies, such as flexible workplace and work hours, to empower employees with greater control over their schedules and better fulfill family responsibilities (60). Moreover, Employee Assistance Programs (EAPs) can provide employee invaluable support through counseling and caregiving services in non-work domains (69). Additionally, offering training program on work-life balance and stress management are also helpful for employee to achieve work-life enhancement (70). Previous studies have also identified that promoting work–family enrichment can benefit both work and family domains. This may be a result of lower job exhaustion (71), better job performance (72), and increased home commitment (73) and family satisfaction (74). However, although improvement in work–family interface has several positive direct effects, the current study emphasizes the importance of managers not overlooking the interaction between the work and family domains. Specifically, some factors of the work–life interface may play a harmonizing role in organizational management. Therefore, managers could provide support and assistance to employees in family-related matters, which is one trait of benevolent leadership (5).

### 5.3 Limitations and future directions

This study has several limitations. First, environmental competitiveness was measured at the individual level by capturing employees’ perception of environmental competitiveness. However, environmental competitiveness can be also assessed at the organizational level and have cross-level effects. Cross-level research offers a comprehensive understanding by integrating individual and organizational perspectives. It identifies how organizational competitive climate influence employees’ outcomes, providing more effective organizational managerial interventions. Therefore, future studies may test the cross-level hypothesis model to investigate the effects of organizational competitive climate on individuals.

Second, due to crossover effect of crossover-spillover model (41) is emphasizing on the interpersonal emotional contagion, this study only explained the spillover effects of individual differences. However, Bakker and Demerouti (41) also highlights interpersonal emotional contagion within groups. By not addressing within-group crossover effects, this study may have overlooked important mechanisms of emotional contagion among group members. Therefore, the effects and mechanisms of within group crossovers should be examined further in future studies.

Thirdly, this study only controlled for demographic variables as common control variables. However, previous research has pointed out the importance of considering additional potential variables such as job stress and job satisfaction, which can also have significant effects on employee psychological perceptions and behaviors in the workplace (45). Thus, future studies could control for additional potential variables that may influence employees’ psychological perceptions and work outcomes.

Fourth, the present study only examined the mediating role of job crafting between perceived environmental competitiveness and mental health. Other potential mechanisms can be investigated in the future. For instance, Oubrich et al. (7) posit that a competitive work environment can lead to employee knowledge hiding behaviors. Moreover, Venz and Nesher Shoshan (75) found that knowledge hiding is negatively correlated with psychological strain. Therefore, future studies may explore the mediating role of knowledge hiding in the relationship between competitive work environment and psychological strain. These future directions could reveal potential mechanisms through which perceived environmental competitiveness impacts employee mental health.

Finally, more work–family interface moderators could be tested in the future. This study only tested the moderating effects of work–family conflict. Future studies could test other factors from domains of work–life or work–family, such as work–family enrichment, work–life balance, or family–work conflict. Specifically, female work–family role conflict (76) is a potential and valuable factor to be investigated, as it addresses the unique challenges faced by women in balancing work and family roles. These moderators could provide valuable insights into how these factors interact with perceived environmental competitiveness to influence employee outcomes across both work and non-work domains.

## 6 Conclusion

This study provides insights into perceived environmental competitiveness that can serve as motivation for employees to engage in job crafting, subsequently enhancing their mental health. We also emphasized how individuals with low work–family conflict can use the effects of perceived environmental competitiveness to improve their overall mental health via job crafting. Our study provides useful insights for managers who wish to foster a healthy, competitive work environment.

## Data Availability

The raw data supporting the conclusions of this article will be made available by the authors, without undue reservation.
